# Superhydrophobic Non-Metallic Surfaces with Multiscale Nano/Micro-Structure: Fabrication and Application

**DOI:** 10.3390/molecules29092098

**Published:** 2024-05-01

**Authors:** Qi Guo, Jieyin Ma, Tianjun Yin, Haichuan Jin, Jiaxiang Zheng, Hui Gao

**Affiliations:** 1 School of Aeronautic Science and Engineering, Beihang University, Beijing 100191, China; guoqi1996@buaa.edu.cn (Q.G.); mznxmjy@buaa.edu.cn (J.M.); yintj330@163.com (T.Y.); jinhaichuan@buaa.edu.cn (H.J.); zhengjx@buaa.edu.cn (J.Z.); 2 Ningbo Institute of Technology, Beihang University, Ningbo 315100, China

**Keywords:** superhydrophobic surfaces, multiscale structures, non-metallic materials, fabrication methods, applications

## Abstract

Multiscale nano/micro-structured surfaces with superhydrophobicity are abundantly observed in nature such as lotus leaves, rose petals and butterfly wings, where microstructures typically reinforce mechanical stability, while nanostructures predominantly govern wettability. To emulate such hierarchical structures in nature, various methods have been widely applied in the past few decades to the manufacture of multiscale structures which can be applied to functionalities ranging from anti-icing and water–oil separation to self-cleaning. In this review, we highlight recent advances in nano/micro-structured superhydrophobic surfaces, with particular focus on non-metallic materials as they are widely used in daily life due to their lightweight, abrasion resistance and ease of processing properties. This review is organized into three sections. First, fabrication methods of multiscale hierarchical structures are introduced with their strengths and weaknesses. Second, four main application areas of anti-icing, water–oil separation, anti-fog and self-cleaning are overviewed by assessing how and why multiscale structures need to be incorporated to carry out their performances. Finally, future directions and challenges for nano/micro-structured surfaces are presented.

## 1. Introduction

A superhydrophobic non-metallic surface refers to surfaces engineered to exhibit an extremely high water contact angle (WCA, θ) with no less than 150° through surface modification techniques [1]; these surfaces play an indispensable role in our daily life and can be found in applications across diverse sectors of daily necessities, architecture, healthcare, textiles, transportation and environmental conservation [2,3,4,5,6,7,8,9,10,11,12,13,14,15].

The concept of surface modification to enable superhydrophobicity is derived from the lotus effect [16]. The upper surface of the lotus leaf possesses an excellent water-repellency property as a result of its well-organized hierarchical structures from the nano to the micrometer scale and a wax-like coating that contributes to a large contact angle and a small roll-off angle. This observation reveals two crucial characteristics for achieving superhydrophobicity on a solid surface, nano/micro-structured topography and a low surface energy [17,18]. Among them, nano/micro-structured topography pertains to the array of concave–convex structures occurring at micro- and nano-scales, resulting in a reduction in the contact area between the liquid and the surface. Meanwhile low surface energy refers to a weak interactive force between surface molecules, making the surface resistant to wetting or covering by other substances. A similar phenomenon, additionally, is also observed on the surfaces of pitcher plants [19], water strider legs [20], rose petals [21], rice leaves [22] and other creatures. Such findings further promote the development and investigation of biomimetic superhydrophobic surfaces because of their outstanding performances and potential applications including, but not limited to, anti-icing [23,24], anti-fogging [25,26] and self-cleaning [27,28].

**Figure 1:**
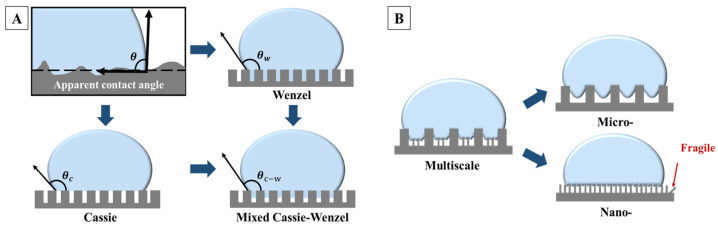
(**A**) The apparent contact angle θ, and a schematic diagram of Wenzel’s model, Cassie–Baxter’s model, and the mixed Cassie–Wenzel model. (**B**) A schematic diagram of the difference between multiscale structures and single micro-structures and single nano-structures.

Such surfaces with multiscale hierarchical structure which possess excellent superhydrophobicity can be theoretically well explained. As early as 1805, Young’s equation [29] described the relationship between WCA and surface energy at the solid–liquid–gas three-phase interface, as shown in Equation (1):

(1) $$\gamma_{LV}\cos\theta_{Y}=\gamma_{SV}-\gamma_{SL}$$

where $\gamma_{SV}$, $\gamma_{LV}$ and $\gamma_{SL}$ represent the free energy per unit area at the solid–liquid interface, liquid–gas interface and solid–gas interface, respectively. $\theta_{Y}$ denotes the Young’s angle. This equation indicates that a lower solid–liquid surface energy results in a higher contact angle. However, the actual surfaces are not absolutely smooth. To better account for surface roughness, the Wenzel model [30] and Cassie–Baxter model [31] were introduced and widely recognized for elucidating the phenomenon of superhydrophobicity on complex structures [32], as illustrated in Figure 1A. In practice, the actual wettability of surfaces lies between the extremes described by the Wenzel and Cassie–Baxter models, referred to as the mixed Cassie–Wenzel model (Figure 1A), where the liquid partially wets the rough surface structure [33]. In such cases, the contact angle can be analyzed by Equation (2).

(2) $$\cos\theta_{C-W}=f_{1}\left(r\cos\theta_{Y}+1\right)-1$$

where $\theta_{C-W}$ represents the apparent contact angle, $f_{1}$ is the proportion of the contact area between the liquid droplet and the rough surface, and r denotes the surface roughness. Building upon the foundation of low surface energy, this model further introduces a key parameter of superhydrophobicity, the small solid–liquid contact area, which can be achieved by constructing multiscale nano/micro-structures on the surface. Multiscale structures, as depicted in Figure 1B, can create more micro cavities and protrusions in contrast to simple rough micro-structures, leading to a smaller solid–liquid contact area. This aids in capturing more air on the surface, forming micro air pockets. Meanwhile, nano structures with high aspect ratios are mechanically fragile [34], whereas micro-scale protrusions have higher mechanical stability [35]. A combination of the two can create a surface with excellent superhydrophobicity and high robustness, offering more possibilities for surface structure design [36,37].

Superhydrophobic modification via multiscale structures is therefore the focus of current research, with a continuous influx of novel findings. The multiscale nano/micro-structure enhances the material surface with a higher contact angle and stronger water-repellency. However, compared with a single structure, the preparation process of nano/micro-structures is more complex, requiring precise processing technology and material design. Researchers have developed various methods such as the sol–gel technique, the etching method, etc., to fabricate concave–convex structures occurring at micro- and nano-scales for different applications. Substrates with diverse material compositions can be designed and modified with multilevel nano/micro-structures to achieve superhydrophobicity. Non-metallic materials offer advantages such as eco-friendliness and lower costs compared to metallic materials. The demand for superhydrophobic non-metallic surfaces is increasing in various fields, including construction, automotive, aerospace and medical industries. For instance, superhydrophobic modification of transparent materials like inorganic glass or organic polymers can prevent fogging, improve self-cleaning ability and enhance safety of use [38]. In applications involving composite materials, such as wind turbine blades and aircraft wings, using superhydrophobicity to prevent icing has become a recent research focus [39,40]. In the field of oil–water separation, non-metallic materials are gradually replacing metallic ones due to their excellent corrosion resistance and environmental friendliness, becoming the topics of current interest [41,42]. It is evident that there is a high demand for superhydrophobic non-metallic surfaces across different industries. Despite this demand, there exists a dearth of systematic summarization of methods for superhydrophobic surface modification with multiscale nano/micro-structures in non-metallic materials.

**Figure 2:**
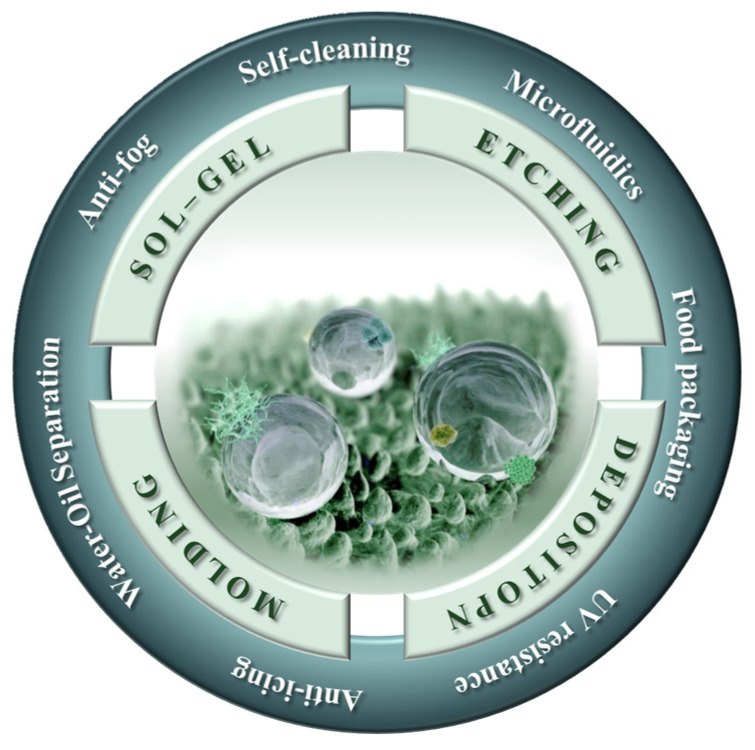
A schematic diagram illustrating the different preparation methods and applications of the superhydrophobic non-metallic surfaces with nano/micro(multiscale)-structures discussed in this review [43].

Hence, this review systematically summarizes the latest research, the common methods and the application prospects of the superhydrophobic non-metallic surfaces with nano/micro(multiscale)-structures, as shown in Figure 2, which will make an important contribution to the production and utilization of superhydrophobic surfaces on non-metallic materials, and the preparation of multiscale structures on the surface of non-metallic materials.

## 2. Preparation Methods of Nano/Micro-Structured Superhydrophobic Surfaces

To achieve superhydrophobic surfaces with unique wettability and structures, a variety of techniques have been employed. Previous research has mainly concentrated on enhancing superhydrophobicity. However, with the development of technology, besides superhydrophobicity, people have started to pay more attention to the overall performance of superhydrophobic surfaces, such as robustness and large-scale preparation. Consequently, higher demands are being placed on the preparation technology. In this section, the preparation techniques of non-metallic surfaces with multiscale nano/micro-structures are mainly concerned. Various common techniques are introduced respectively, which can be mainly divided into physical and chemical methods [44,45]. Physical methods include etching, molding and physical vapor deposition (PVD) to form microscopic and nano-scale structures on the material surfaces through precision machining or by using a stencil [46,47]. Chemical methods include the sol–gel method, chemical vapor deposition (CVD) and electrochemical deposition (ECD), which change the surface energy and structure by depositing or synthesizing compounds on the surfaces [48,49,50]. These methods can be applied individually or in combination to meet the needs of multiscale nano/micro-structured superhydrophobic surfaces for different applications.

### 2.1. Sol–Gel Method

**Figure 3:**
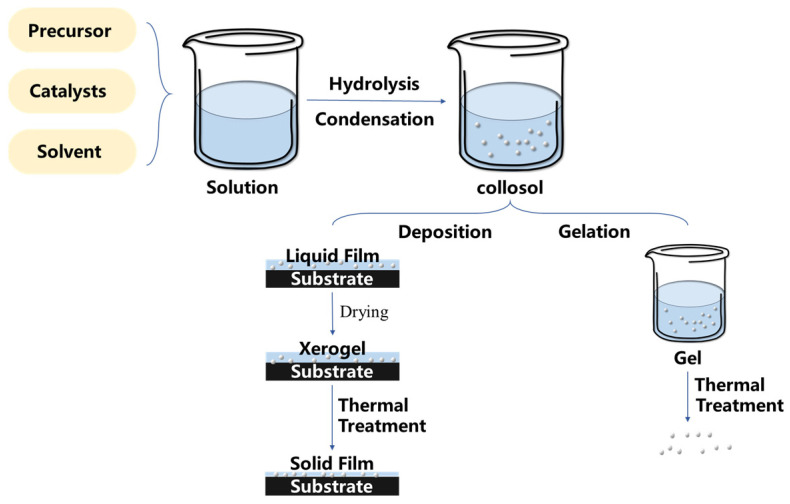
A diagram of the main steps in a typical sol–gel process.

The sol–gel method stands as a prevalent technique for the preparation of wet chemical materials [51]. In this process, the precursor undergoes hydrolysis in the liquid phase, leading to the formation of a gel system through the condensation polymerization of colloidal particles [52,53]. Then, the nano-particles in the gel system can be adsorbed on the substrate, forming a thin film combined with the surface of the substrate material [54,55,56]. Another way is to manage the gel system by using heat treatment to obtain treated nano-particles [57] and then cover the surface by using physical methods such as the spraying method or the dip-coating method. The schematic diagrams of the two routes are shown in Figure 3. In addition, the superhydrophobic properties can also be further enhanced or functionalized through chemical modification. The sol–gel method offers a well-established route for creating superhydrophobic surfaces on non-metallic substrates [58]. Despite limitations in morphology controllability through spraying or dipping [59,60], it still provides a versatile platform for doping new materials and is compatible with various substrates [61,62,63], enhancing the possibilities of the surface design. Nonetheless, due to its numerous advantages, the sol–gel method remains one of the important methods for preparing superhydrophobic surfaces with multiscale structures on non-metallic materials.

**Figure 4:**
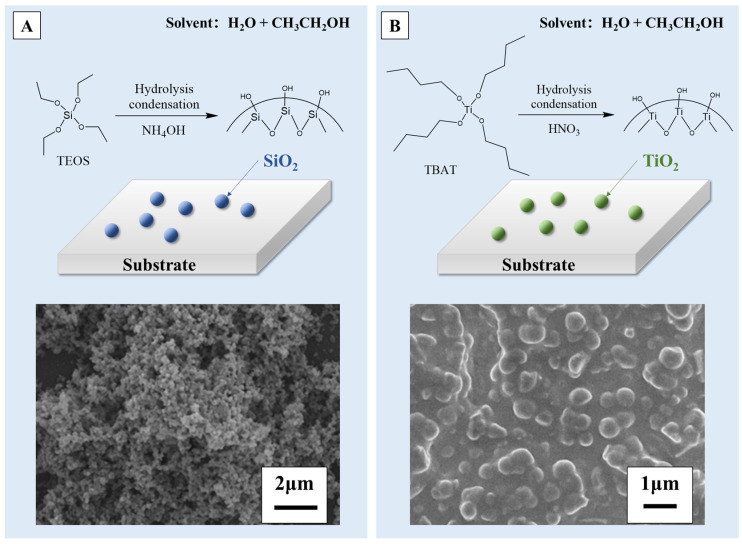
A schematic illustration and scanning electron microscope (SEM) image of (**A**) SiO_2_ [74,75] and (**B**) TiO_2_ coatings [76] prepared by the sol–gel method. Reprinted with permission from [74,75,76], Copyright 2021 MDPI, Copyright 2019 MDPI, Copyright 2020 Elsevier, respectively.

Superhydrophobic surfaces with different non-metallic compositions can be facilely produced by the sol–gel method. For example, silica and titania coatings are common materials used for superhydrophobic surface modification through the sol–gel method [64,65,66,67,68]. By means of the classical Stöber process [52,69,70,71,72,73], as depicted in Figure 4A, Heiman-Burstein et al. [74] achieved a superhydrophobic coating by modifying silica nano-particles through the in situ addition of long-chain alkyl silane co-precursors, in combination with tetraethyl orthosilicate (TEOS). They discovered that when the alkyl length exceeded ten carbons, a superhydrophobic coating could be achieved, showcasing a raspberry-like hierarchical morphology. The direct condensation of silica nano-particles (NPs) on the substrate surface results in covalent bonding between the TEOS/alkyl silane systems and compatibilized (oxygen-treated) substrates. This covalent bonding facilitates the attachment of the silane-treated particles to the substrate, enhancing the coating’s durability. Wang et al. [75] also prepared silica modified cellulose fibers via a modified Stöber method, obtaining a hierarchical superhydrophobic surface with a WCA up to 151.3°. As for the titania coating, Hu et al. [76], inspired by the reversible swelling ability of cured rubber, immersed the swollen silicone rubber (SR) in a tetrabutyltitanate (TBT) solution (precursor of sol–gel). The precursor moved into the SR and came into contact with the catalyst, resulting in the generation of TiO_2_ particles in the crosslinking network of SR. The TiO_2_ particles grew gradually and formed a texture of multiscale roughness on the SR surface, which is shown in Figure 4B, with a WCA and a roll-off angle of 158.6° and 6.5°. The embedding of TiO_2_ particles in SR enhanced the mechanical durability of the superhydrophobic surfaces compared to samples prepared by conventional sol–gel methods. Nasiri Khalil Abad et al. [77] also designed a Cd-Si co-doped TiO_2_ thin film, in which titania nanoparticles were synthesized by the sol–gel method. The results illustrate that the increasing calcination temperature triggered the agglomeration of particles, which induced a WCA of nearly 168° on the surface.

The gel can also serve as a functional adhesive material, adhering other nano-particle fillers to the surface [78,79]. The combination of the two forms of nano/micro-structures induced superhydrophobicity on the surface. Zhou et al. [80] prepared the siloxane zwitterionic compound (GPAC) through a modified sol–gel method, mixing it with carnauba wax micro-particles to obtain a clear colloidal suspension (W-GPAC). The wax displayed a spherical structure with large pores throughout the surface. Meanwhile, the GPAC sol showed a wrinkled structure, and the “close-packed” layered structure could be observed in the W-GPAC composite, which could be beneficial due to its durability and good mechanical property. Experimental tests indicate that the surface’s WCA can reach 170°, demonstrating outstanding superhydrophobicity. Meanwhile, the amine group within the W-GPAC sol can form hydrogen bonds with the substrate, while 3-Glycidyloxypropyltrimethoxysliane(KH-560) facilitates excellent adhesion through a coupling reaction during the coating’s curing process. These mechanisms collectively enhance the adhesion between the coating and the substrate. Likewise, Patra et al. [81] used silica as a nano-particle filler, mixed it in the gel system with 1*H*,1*H*,2*H*,2*H*-perfluoro-octyltriethoxysilane (FTS), and deposited it onto the substrate to form nano/micro-structures.

In recent years, significant advancements in achieving high superhydrophobicity have been demonstrated by superhydrophobic coatings prepared via the sol–gel method. Researchers in the field are now gradually exploring methods to enhance the robustness and durability of these coatings, such as physical methods (e.g., embedding) and chemical methods (e.g., bonding). This provides a new idea for subsequent research on multiscale superhydrophobic surfaces and further improves the reliability of superhydrophobic coatings.

### 2.2. Etching Methods

Etching methods are a common technique utilized for the preparation of multiscale-structured superhydrophobic surfaces [82] which can precisely control the surface structure and morphology by etching the substrate, such as through chemical etching, laser etching, electrochemical etching and plasma etching [83,84,85]. To achieve superhydrophobicity, the etched substrates are then modified by low-surface-energy materials [46]. It should be mentioned that chemical etching and electrochemical etching are mainly applied to metallic materials, so they are not discussed in this work. In contrast, physical etching methods such as laser etching and plasma etching are generally used for non-metallic materials, so they are discussed in detail as follows.

#### 2.2.1. Laser Etching Method

**Figure 5:**
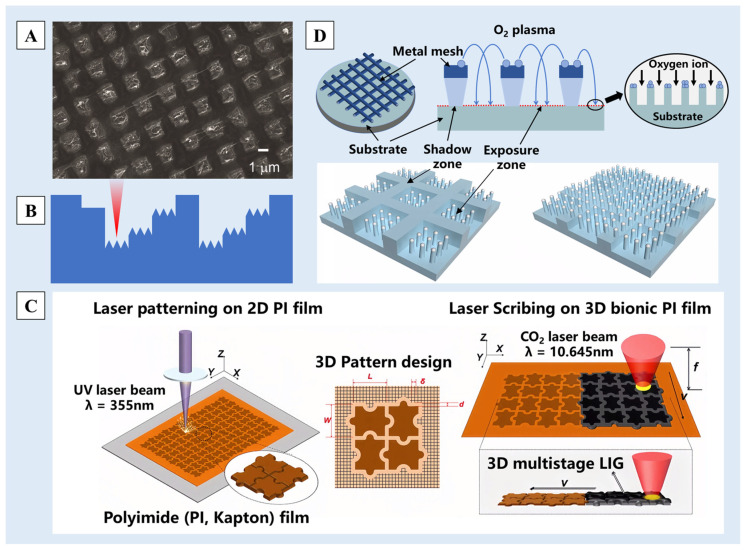
(**A**) A SEM image of the graphene structure produced with 0.8 W laser power [88]. (**B**) The stepped structure produced by adjusting the laser etching times [92]. (**C**) A schematic illustration of a jigsaw-like laser-induced graphene (LIG) fabricated by laser patterning on a pure PI film and then by laser scribing on a three-dimensional (3D) bionic PI film [93]. (**D**) Illustrations of a hybrid patterning process assisted by a metal mesh overhang showing two characteristic etching masks [96]. Reprinted with permission from [88,92,93,96], Copyright 2019 SciEngine, Copyright 2018 John Wiley and Sons, Copyright 2022 Springer Nature, Copyright 2020 Springer Nature, respectively.

The laser etching method [86] works by directing a high-energy laser beam onto the material surface, causing a series of reactions such as melting and vaporization under the action of photoelectric or photothermal effects and finally forming rough multiscale nano/micro-structures. Due to the excellent controllability of laser etching, this method can be used to etch nano/micro-structures on thin surfaces [87], such as the oxidized graphene film [88] and the polydimethylsiloxane (PDMS) film [89]. For example, when the oxidized graphene film was overlaid on a fabric surface and subjected to double-laser interference ablation, graphene nano-structures were generated by deoxygenation at the ablated locations (Figure 5A). This, in conjunction with the larger micrometer-scale structures of the underlying fabric, forms a nano/micro-multiscale graphene surface, resulting excellent superhydrophobicity [88].

The size and shape of multiscale nano/micro-structures can be controlled by changing the laser parameters or by controlling the position, energy and frequency of the laser etching. Thus, columnar, channel, stepped and other different pattern structures are prepared on the substrate surfaces [89,90,91]. Fang et al. [92] used femtosecond laser ablation technology to construct a micro-groove array structure on the surface of PDMS and further introduced nano/micro-step structures (Figure 5B) by adjusting the laser etching times at different positions. Wang et al. [93], inspired by the nano/micro-structures on the surface of Oxalis corniculata Linn., used an ultraviolet (UV) laser (355 nm) and CO_2_ laser (10.64 μm) to etch polyimide (PI) films sequentially, and a jigsaw-like micro-structure was obtained (Figure 5C). The structure and porous graphene constituted the three-dimensional multiscale structure of the surface, and a multiscale biomimetic graphene surface was obtained.

#### 2.2.2. Plasma Etching Method

Plasma etching utilizes high-frequency glow discharge reactions, activating reaction gases into reactive particles that diffuse to the etching site. Upon inter-reacting with the etched material, volatile by-products are generated and eliminated subsequently, achieving the purpose of etching [45,94,95]. Compared to laser ablation methods, plasma etching has higher dimensions but lower precision, often requiring the assistance of surface molds or oxide layers [35]. Ko et al. [96] proposed a one-step approach using metallic mesh masking. Through a plasma selective etching process assisted by double-scale etching mold, they prepared a layer-structured superhydrophobic surface (Figure 5D). By controlling the gap distance between the substrate and the metallic mesh, the dual-scale etching masking effect was achieved, achieving a water contact angle of up to 164.1°. Zhang et al. [97] developed superhydrophobic cotton fabric through a process involving oxygen plasma etching followed by the application of a hydrophobic suspension containing SiO_2_ nano-particles via spraying. The SiO_2_ nanoparticles were deposited on the fiber, which together with the uniform strip grooves created by plasma etching contributed to a dual-scale rough structure on the fabric surface.

Etching methods have high processing precision and good controllability, which can accurately control the surface microscopic shape and prepare complex multiscale nano/micro-structures [85,98], thus improving the superhydrophobicity, and they are suitable for a variety of non-metallic materials. However, the equipment is expensive and the processing cost is high [50], which is not suitable for industrial large-scale production. Among them, plasma etching is more efficient than laser etching, while laser etching is more environmentally friendly [46].

### 2.3. Molding Method

The molding method [99,100,101] involves depositing the target material on the natural or artificial template to reproduce the rough structure of the template. As early as 2009, researchers attempted to use the molding method to prepare superhydrophobic surfaces and successfully obtained polymer surfaces with a contact angle of 167° [102]. In recent years, the micro-structure of superhydrophobic surfaces prepared by the molding method has gradually developed from single-scale roughness to a double-scale micro-structure to enhance the mechanical durability of the surface. And the multiscale superhydrophobic surface can be produced on a large scale. The key with the molding method is the preparation of defect-free molds, which can be typically achieved through methods such as etching or 3D printing.

**Figure 6:**
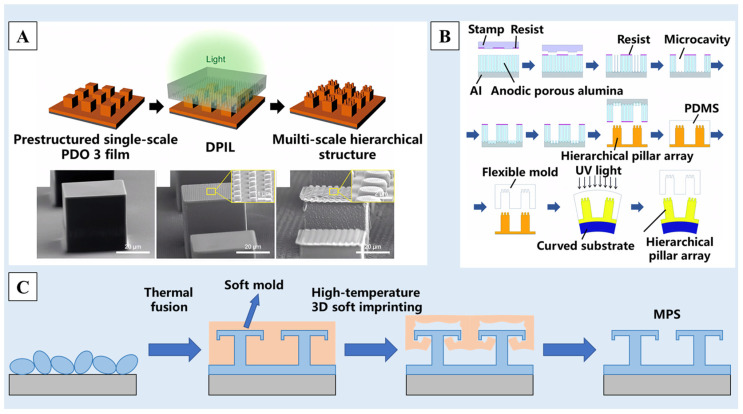
(**A**) Multiscale hierarchical structures formed by DPIL [105]. (**B**) The states of a droplet on surfaces of different roughness: micro-papillae, sub-mm papillae and hybrid papillae [106]. (**C**) A schematic illustration of the fabrication method consisting of thermal fusion and high-temperature 3D soft imprinting [108]. Reprinted with permission from [105,106,108], Copyright 2017 American Chemical Society, Copyright 2022 RSC Publishing, Copyright 2023 Elsevier, respectively.

Photolithography [103] and chemical etching [104] are common methods for making molds. The specific scale molds are produced by photoetching or chemical etching, and then, the required raw materials are affixed to the mold surface, resulting in the creation of a superhydrophobic surface with a nano/micro-structure. As shown in Figure 6A, Choi et al. [105] proposed a directional photofluidization imprint lithography (DPIL) method, in which polydisperse orange 3 (PDO 3) and bisphenol A-type epoxy resin were used as raw materials to make thin-film materials and then silicon molds of different scales were prepared by photolithography. The raw materials were polymerized and cured in different molds many times, following by being bonded to the surface of the film to form the multiscale nano/micro-structure. As shown in Figure 6B, a two-step template approach was used to create flexible molds with hierarchical nano/micro-structures on the surface, with anodic porous alumina serving as a starting material. On the surfaces of tubular substrates and convex lenses, ordered pillar arrays with hierarchical nano/micro-structures may be created by photo-nanoimprinting utilizing the acquired flexible molds [106]. In recent years, with the development of 3D printing technology, researchers have tried to use this method to make molds [107]. Li et al. [108] prepared a silicone hot film with elasticity and shape memory at a high temperature in the 3D printing mold, and made T-shaped grooving on it. The perfluoropolymer material coated on any surface was pressed with the mold at 280 °C, resulting in a multiscale superhydrophobic structure of the monolithic perfluoropolymer surface (MPS) with a contact angle up to 160° (Figure 6C).

**Figure 7:**
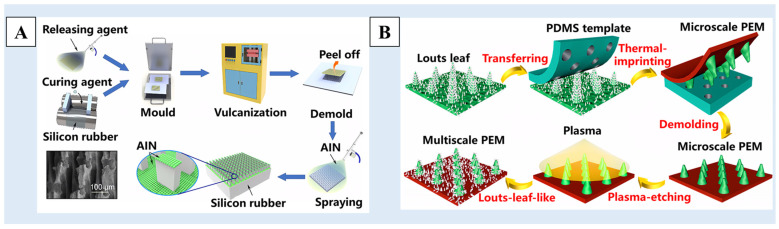
A schematic of the preparation process of (**A**) a multilevel rough-structured superhydrophobic surface using stainless steel mesh as a template [109] and (**B**) the superhydrophobic membrane with a multiscale structure using lotus leaf as a template [112]. Reprinted with permission from [109,112], Copyright 2023 American Chemical Society, Copyright 2023 American Chemical Society, respectively.

In addition to the above methods for preparing templates, some researchers have directly used existing materials with rough surface structures as templates, such as stainless steel mesh [109], woven fiber cloth [110], etc. As shown in Figure 7A, He et al. [109] used stainless steel mesh as a template and silicone rubber as a raw material, and transferred the structure of the stainless steel mesh to the surface of the silicone rubber through the template method to build a surface structure. Then, the surface coating structure was constructed by spraying aluminum nitride (AlN) particles on the surface of silicone rubber. A multiscale rough structure surface with excellent superhydrophobicity was obtained. Some people have also used natural superhydrophobic materials, such as lotus leaves [111,112,113], rose petals [114], etc. As show in Figure 7B, using the lotus leaf as a template, Li et al. [112] imprinted the micro-scale pillars of the lotus leaf onto the PDMS resin. Then, the micro-scale pillar structure was transferred to the proton exchange membrane (PEM) surface by using a thermal imprinting procedure to obtain a surface with a micron structure. Finally, a secondary nano-structure was formed by using etching technology, and the surface with a multiscale nano/micro-structure was obtained.

The molding method can also be applied to surfaces with complex shapes. It is relatively simple and does not require the precise control of reaction time [115,116]. The emphasis is on the quality of the molds, as a high-quality mold can produce multiple samples, enabling large-scale batch production of superhydrophobic surfaces and reducing manufacturing costs and time [106,117,118]. Nonetheless, the high cost of mold preparation and the risk of damaging surface micro-structures during the mold–material separation process, coupled with the insufficient precision of most mold fabrication methods, pose challenge in achieving a one-step formation of nano/micro-structured multiscale superhydrophobic surfaces. We still need other methods to enrich the nano-structure of the surface produced by molding.

### 2.4. Deposition Methods

There are many deposition methods for preparing superhydrophobic surfaces with multiscale structures, among which physical vapor deposition (PVD), chemical vapor deposition (CVD) and electrochemical deposition (ECD) can be used to construct structures on non-metallic surfaces [50,119,120]. When the superhydrophobic modification is carried out by using deposition methods, two steps are generally needed: one is the design of the surface nano/micro-structure; the other is the modification by low-surface-energy substances.

#### 2.4.1. Physical Vapor Deposition (PVD)

**Figure 8:**
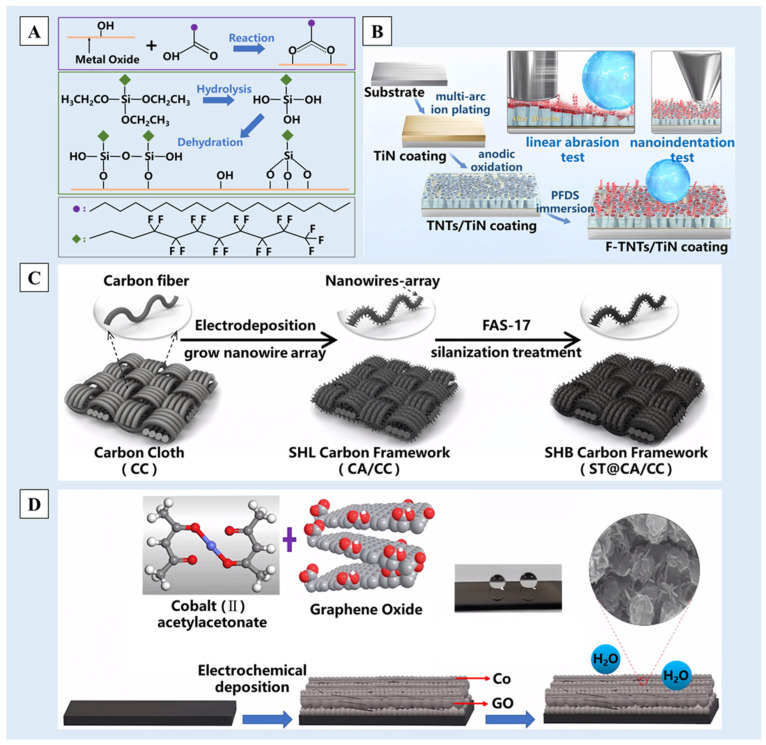
A schematic diagram of the preparation process of (**A**) the self-assembled films on the SDC coating surface by using stearic acid and FAS in the PVD method [123], (**B**) the preparation of an F-TNTs/TiN composite coating by using the PVD method [124], (**C**) the preparation of photothermal superhydrophobic materials by using the ECD method [135] and (**D**) the preparation of the superhydrophobic G-Co/a-C:H film by using the ECD method [136]. Reprinted with permission from [123,124,135,136], Copyright 2017 Springer Nature, Copyright 2023 Elsevier, Copyright 2021 American Chemical Society, Copyright 2018 John Wiley and Sons, respectively.

PVD is a process in which solid materials are atomized or vaporized, and deposited on the substrate surface to form thin films with thicknesses ranging from atomic layers to several micrometers [121,122]. PVD processes are often conducted in a vacuum, plasma or electrolytic environment, which can minimize gas contamination during the deposition process. In accordance with the deposition steps, Li et al. [123] employed a plasma spray system to deposit Samaria-doped ceria (SDC) at a low pressure on a ceramic substrate. And the surface was secondarily modified by stearic acid or 1,1,2,2-tetrahydroperfluorodecyltrimethoxysilane (FAS) (Figure 8A). Gao et al. [124] employed multiarc ion plating to prepare ultrathin titanium-based hard coatings on the substrate, which were then modified by perfluorodecyltriethoxysilane (PFDS). In both cases, multiscale nano/micro-structured superhydrophobic surfaces with a water contact angle of 150° could be achieved (Figure 8B).

#### 2.4.2. Chemical Vapor Deposition (CVD)

The main difference between chemical vapor deposition, chemical bath deposition and electrochemical deposition is the different environment in which the chemical reaction takes place. CVD mainly uses one or several vapor compounds or elements containing thin-film elements to chemically react together on the surface of substrate to produce a thin film [45,125]. Commonly used deposition materials include carbon nanotubes [126,127], SiO_2_ [128,129], TiO_2_ [130], PDMS [131,132] and so on. For example, Tombesi et al. [133] used aerosol-assisted chemical vapor deposition (AACVD) to fabricate SiO_2_ nano-particle films with dual-scale roughness on glass substrates. The films had excellent superhydrophobicity and transparency.

#### 2.4.3. Electrochemical Deposition (ECD)

Electrochemical deposition is a coating technology in which a redox reaction occurs under the action of an applied electric field [120,134]. Therefore, the deposited surface needs to have a certain conductivity, and carbon-based materials and silicon-based materials are usually used as substrates for electrochemical deposition in the field of non-metals. For example, Xie et al. [135], after hydrophilic treatment of the carbon cloth surface, deposited a polypyrrole nano-wire array on the surface through electrochemical deposition, carbonized it at a high temperature and finally modified it with 1*H*,1*H*,2*H*,2*H*-perfluorodecyltrimethoxysilane (FAS-17) with a low surface energy to obtain a superhydrophobic photothermal carbon-based material with a cascade nano/micro-structure (Figure 8C). When a silicon-based material is used as the substrate, the surface oxide layer needs to be removed in order to ensure the conductive effect. Using methanol as a carbon source, graphene-doped metal cobalt [136] or metal nickel [137] was used for electrochemical deposition on the surface of silicon to obtain doped graphene/metal carbon films (Figure 8D). Under SEM, it was observed that graphene and metal combined to form a nano- and multiscale composite interface, thus forming a well-structured nano/micro-structure.

The deposition method for preparing multiscale nano/micro-structured superhydrophobic surfaces is simple, reproducible, cost-effective and well developed [44,138]. It does not require complex equipment and is more suitable for large-area surface treatment [50,120,139]. Moreover, compared to the sol–gel method, it is easier to control the surface morphology. But the deposition method is constrained by the substrate surface. For example, electrochemical deposition is only applicable to conductive non-metallic materials, and its durability is inferior to etching methods.

### 2.5. Other Methods

**Figure 9:**
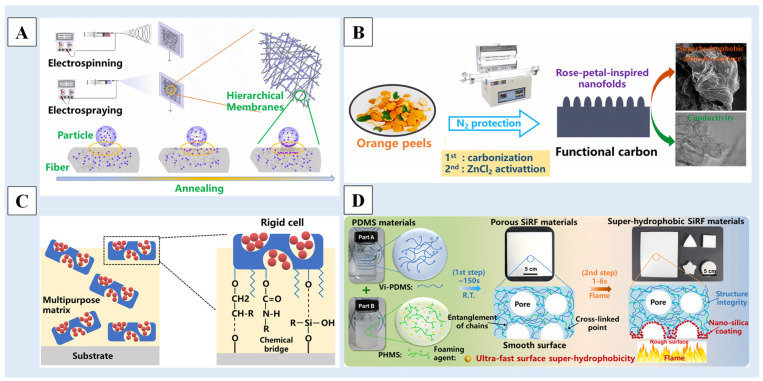
A schematic diagram of (**A**) the preparation of hierarchical composite membranes by using sequential electrospinning and electrospraying [140], (**B**) the conversion of orange peels to the conductive and superhydrophobic carbon [156], (**C**) the cellular design [157] and (**D**) the preparation process of superhydrophobic SiRF materials [158]. Reprinted with permission from [140,156,157,158], Copyright 2020 Elsevier, Copyright 2023 Elsevier, Copyright 2023 Springer Nature, Copyright 2021 American Chemical Society, respectively.

In addition to the above-discussed sol–gel method, molding method, etching method and deposition method, there are some other methods that can prepare multiscale-structured superhydrophobic surfaces of non-metallic materials, such as the electrospinning method [140,141,142,143,144,145], the self-assembly method [146,147,148,149,150], the spraying method [151,152,153], the dip-coating method [154,155] and so on. As shown in Figure 9A, Gan et al. [140] used a sequential electrospinning and electrospraying method to fabricate composite membranes of polystyrene-block-poly (ethylene-*co*-butylene)-block-polystyrene (SEBS) and fluorinated polyhedral oligomeric silsesquioxane-block-polystyrene (FPOSS-PS). The composite membranes with hierarchical geometries and low-surface-energy modifications had excellent superhydrophobicity, flush resistance and anti-adhesion properties.

In addition, there are some newly invented methods as well. Chen et al. [156] pressed orange peel into a powder after drying, carbonization at a high temperature, mixing with zinc chloride, grounding and treatment at a high temperature to obtain superhydrophobic/superoleophilic carbon derived from orange peel, which has a layered structure with a nano-fold surface and an ordered graphene layer, and has excellent electrical conductivity and excellent oil–water separation characteristics (Figure 9B). Gu et al. [157] proposed a cell composed of hard porous diatomite micro-shells and releasable nano-seeds, which can simultaneously confer the multiphase repulsion and ultralong effectiveness of superhydrophobic coatings without requiring a complex structure and manufacturing process (Figure 9C). Zhang et al. [158] created a mechanically strong and superhydrophobic surface on PDMS foam by using a flame-induced pyrolysis (FIP) strategy. It only took 1~6s in the ultrafast FIP process to build a strong special wavy rough nano/micro-structure on the surface of the silicone rubber foam (SiRF) material to achieve superhydrophobic surface characteristics (Figure 9D).

## 3. Application

The technology of multiscale superhydrophobic modification of non-metallic surfaces is rapidly gaining prominence, and researchers are making unremitting efforts to achieve innovative applications in various fields, including architecture, transportation, healthcare and energy. Here, we summarize several representative applications of nano/micro-multiscale superhydrophobic modifications of non-metallic surfaces, including pollution-resistant, self-cleaning, microfluidics and anti-icing applications, which can demonstrate its importance and widespread applicability across multiple fields, and offer readers a comprehensive view of this technology for a better understanding [49,50,120,159,160,161].

### 3.1. Anti-Icing

Ice accretion on surfaces tends to cause equipment overload and operational damage. Furthermore, the excessive accumulation of ice, followed by the shedding of ice, may result in abnormal service conditions, potentially causing severe safety incidents and economic losses [162]. These disasters occur in numerous fields, such as aviation, power production, building construction and transportation [163,164,165,166,167]. In view of these security risks and energy waste issues, the superhydrophobic modification of material surfaces has become a potential solution to anti-icing issues in recent years [168,169]. The characteristics of a low surface energy, high contact angle and low roll-off angle exhibited by superhydrophobic surfaces can effectively prevent the condensation of water vapor from the air on the surface of equipment and expedite the rolling of water droplets, thereby preventing or delaying icing [163,170,171].

**Figure 10:**
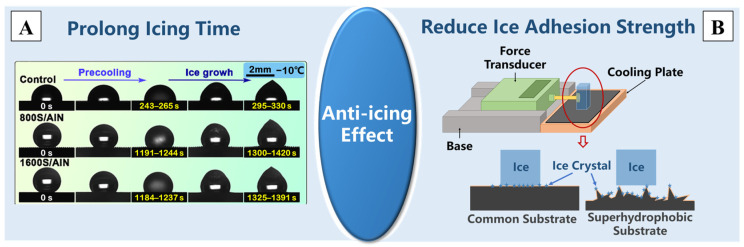
The two aspects of the improvement in the anti-icing effect due to superhydrophobic modification: (**A**) the prolongation of the icing time [109] and (**B**) the reduction in the ice adhesion strength [173,174]. Reprinted with permission from [109,173,174], Copyright 2023 American Chemical Society, Copyright 2021 Springer Nature, Copyright 2019 Elsevier, respectively.

The icing time and ice adhesion strength are two important indicators for evaluating the anti-icing performance of superhydrophobic surfaces [172]. Numerous studies have confirmed that non-metallic superhydrophobic surfaces with nano/micro-structures can prolong the icing time and reduce the ice adhesion strength [173,174], and superhydrophobic surfaces with different nano/micro-sizes exhibit varying delaying effects on icing time [175]. He et al. [109] prepared a multiscale superhydrophobic surface on rubber by using the molding method, and the icing time increased by 3.6 times at −10 °C, as is shown in Figure 10A. Treating carbon fiber-reinforced polymer (CFRP) and polymethyl methacrylate (PMMA) with laser etching could also produce superhydrophobic surfaces with nano/micro-structures, and the test results of icing delay time further support that superhydrophobic surfaces can extend the ice formation time by more than three times [176,177,178]. Wang et al. [179] also prepared a superhydrophobic coating on glass slides by using the spraying method, reducing the ice adhesion strength from 1473 ± 74 kPa for the substrate to 194 kPa for the coating, which can be tested by the device depicted in Figure 10B.

Usually, multiscale superhydrophobic surfaces are combined with other anti-icing or de-icing methods to further enhance the capability of ice prevention, such as the photothermal method [135,180,181,182] and the electrothermal method [155,183]. When combined with thermal methods, the superhydrophobic layer can remain unfrozen for a long time at a low temperature or cause the ice layer to melt into liquid and rapidly detach in order to prevent recondensation.

### 3.2. Water–Oil Separation

**Figure 11:**
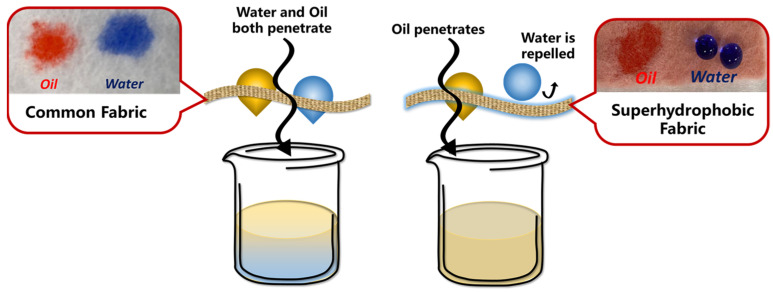
The principle and experimental results of water–oil separation by using superhydrophobic fibers [194] (reproduced from [194] with permission (2022) of the American Chemical Society).

With the rapid development of the energy industry and catering industry, more and more waste oil is produced and discharged into water, resulting in many environmental and water issues [184,185,186]. By adjusting the nano/micro-structure of non-metallic surfaces to create different contact angles with water and oil, efficient water–oil separation can be achieved [1]. The principle of water–oil separation through the use of superhydrophobic materials and a comparison of the contact angle are shown in Figure 11.

Different non-metallic multiscale superhydrophobic materials have been proposed and applied for water–oil separation. Fabrics have a micro-fiber structure, which helps in the formation of small pores and channels for liquid [187]. They are also environmentally friendly and reasonably priced. If the surface undergoes superhydrophobic modification, they are highly suitable as a filtration material for water–oil separation [188,189,190]. Polylactic acid (PLA) is a degradable and environmentally friendly material that can be directly loaded or grown with nano-structures on non-woven fabric made of PLA [191,192]. Coating PLA nanospheres mixed with dioxane on non-woven fabric [193] can also impart excellent hydrophobic and oleophilic properties, completing the water–oil separation process. Other processing methods, such as functionalizing polyacrylonitrile (PAN) non-woven fabric (NWF) with iron hydroxide nano-particles and the in situ deposition of the iron palmitate complex nano/micro-particles, can achieve a water–oil separation efficiency very close to 100% based on the multiscale nano/micro-structures [194]. These validate the potential application of superhydrophobic fabric with multiscale nano/micro-structures in the field of water–oil separation. Polymer membranes [195] and SiC membranes [196,197] can also satisfy the necessary requirements. By using the molding method or the deposition method, the surface can be endowed with nano/micro-structures, exhibiting excellent water–oil separation efficiency. Interestingly, materials commonly found in daily life with loose and porous structures, such as orange peels [156] and cigarette filters [198], can be potential choices for preparing water–oil separation materials.

### 3.3. Anti-Fog

Fogging commonly arises when humid air encounters surfaces or equipment with a lower temperature [199]. For surfaces such as glass or transparent polymers, fogging will reduce light transmission and have adverse effects on daily life and industrial production [200]. Superhydrophobic modification of transparent surfaces, which can achieve anti-fogging, plays a vital role in the areas where it is needed, reducing the impact of surface liquids on performance.

**Figure 12:**
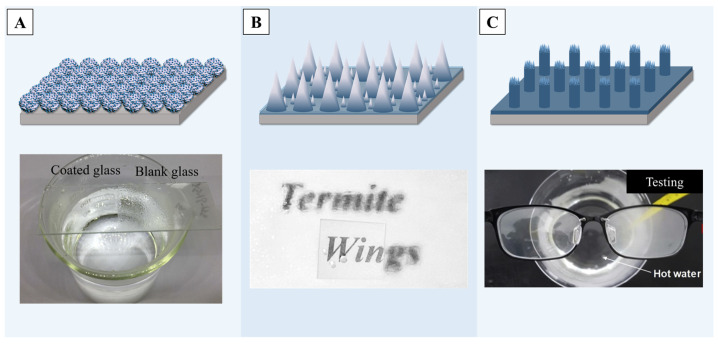
(**A**) Multifunctional nano-coatings of dendritic porous silica nano-particles (DPSNs) @X% TiO_2_ with tunable sizes of TiO_2_ nano-particles (NPs), with the outcome of an enhanced anti-fogging property [203]. (**B**) An illustration of the fabrication procedure for combining colloidal lithography and self-assembly engineered hierarchical conical structures and the performance of anti-fogging [207]. (**C**) The results of hierarchical superhydrophobic polymer (HSP) films fabricated by using photopatterning with a sandpaper template, and the results of anti-fogging tests [210]. Reprinted with permission from [203,207,210], Copyright 2020 Elsevier, Copyright 2023 Elsevier, Copyright 2019 Elsevier, respectively.

Transparent surfaces modified with different materials to form nano/micro-structures for anti-fogging have been widely researched in the past few decades. Nano-sized titanium dioxide [201] or silicon dioxide [26,202] are both good choices as surface modification materials, as they both have a certain degree of light transmission. For example, by loading 2–3 nm titanium dioxide particles onto silica micro-particles, a robust transparent coating with good anti-fogging properties can be prepared [203], as is shown in Figure 12A, and it also has excellent self-cleaning properties. With the help of mold imprinting [204] or laser pulse deposition [205], multiscale titanium dioxide structures can be created on transparent surfaces, endowing the coating with excellent superhydrophobic properties while maintaining a light transmittance of over 90% by adjusting the spacing of the nano/micro-structure, achieving a transparent surface with anti-fogging functions. Other materials like bio-inspired ZnO micro-spheres [25] or ZnO nano-particles [206] can also be employed for surface modification to achieve an exceptional light transparency and anti-fog performance of transparent superhydrophobic surfaces.

It should be mentioned that employing etching or molding methods can endow the transparent materials’ own surface with superhydrophobicity. For example, it was reported that a method combining colloidal lithography and self-assembly engineered hierarchical conical structures achieved superhydrophobicity, which provided a WCA of 175.3° and low contact angle hysteresis (2.7°) [207]. Figure 12B illustrates the anti-fog performance of this surface, showing that even under high-humidity conditions, the text below remains clearly visible compared to the untreated surface. Solidifying the transparent materials on molds with multiscale nano/micro-structures, directly forming films with superhydrophobic properties, can also achieve surfaces with an excellent anti-fog performance [208,209]. A previous report demonstrates that UV-curable polymers can be initially cured into a film on a transparent surface and then treated by multiple curing steps to generate nano/micro-level roughness, achieving superhydrophobicity with a contact angle of 172°. Lenses treated by such materials, as shown in Figure 12C, can still maintain good transparency in high-temperature water vapor environments, showing excellent anti-fog properties [210].

### 3.4. Self-Cleaning

**Figure 13:**
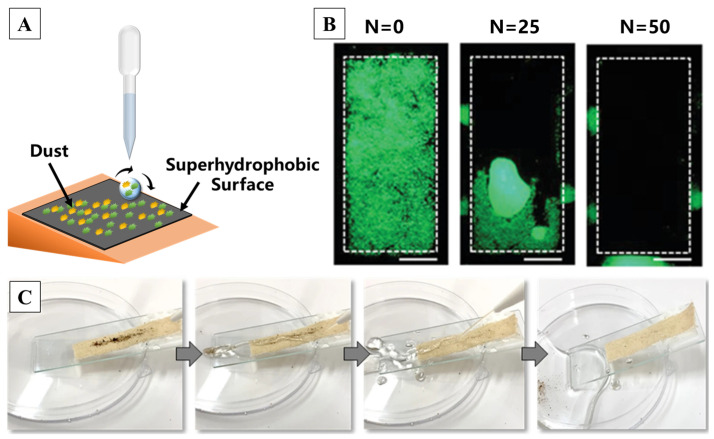
(**A**) A schematic diagram of the self-cleaning test setup [216]. (**B**,**C**) A quantitative and qualitative demonstration of superhydrophobic surface self-cleaning capabilities [216,219], respectively. (N is the number of droplets. Scale bars: 1 cm.) Reprinted with permission from [216,219], Copyright 2022 John Wiley and Sons, Copyright 2021 Springer Nature, respectively.

There is an old saying in China which is as follows: “Lotus unsullied from mud, wash clean without demon”. A surface which has self-cleaning properties can clean its surface itself without any external source, while superhydrophobic surfaces provide a self-cleaning function for pollutants due to its low roll-off angle [59,211,212,213,214]. Briefly, self-cleaning is achieved through the combination of rainwater and a specific tilt angle when there is dust, microorganisms and other stains deposited on the surface of a superhydrophobic coating. In the laboratory, the self-cleaning process can be simulated by dripping water onto the inclined sample surface, as shown in Figure 13A. Such self-cleaning superhydrophobic surfaces find versatile applications in daily life, including car windshields, windows, glass doors, skyscrapers, solar panels, fabrics, sports shoes, metals, paper, sponges, wood, marble and so on [160,215]. It was reported that a superhydrophobic self-cleaning surface based on two types of silica nano-particles with distinct morphologies can remove the fluorescent particles accumulated on the surface due to rolling water droplets [216], as shown in Figure 13B. Constructing multiscale structures on fabrics can also help to achieve self-cleaning functionalization, which can reduce detergent waste, time and labor [217,218]. Pakdel et al. [219] used TiO_2_ particles in the hydrothermal method, followed by nitrogen doping to obtain N-doped TiO_2_, which resulted in flower-like structures on the surface. Through a facile dip-coating method, the particles composed of PDMS were applied on the cotton fabric, forming a nano/micro-structure with commendable self-cleaning properties, as illustrated by Figure 13C.

### 3.5. Other Applications

**Figure 14:**
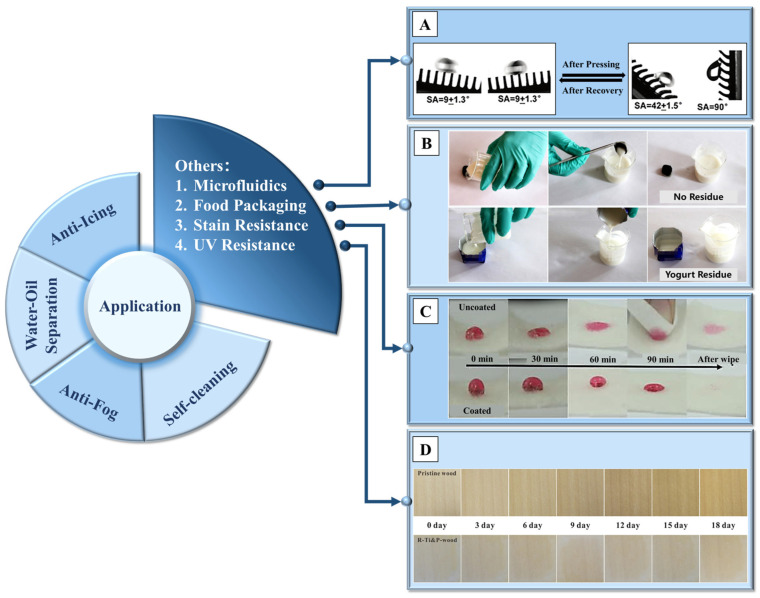
A summary of the non-metallic superhydrophobic surface, with the test result of (**A**) controlling the micro-liquid flow direction due to the directional superhydrophobic surface [224]; (**B**) the residue of yogurt after pouring, compared with commercial packaging [86]; (**C**) the stain resistance of pristine and silica nanoparticle-coated cashmere fabrics [220]; and (**D**) the pristine wood and coated wood within 18 days (taken every 3 days) after UV irradiation [67]. Reprinted with permission from [67,86,220,224]. Copyright 2021 Springer Nature, Copyright 2021 Springer Nature, Copyright 2020 Elsevier, Copyright 2020 American Chemical Society, respectively.

Due to the diverse types and wide range of applications of non-metallic materials, in addition to the four main directions mentioned above, superhydrophobic non-metallic surfaces can also be used in other applications such as microfluidics, food packaging, stain resistance [220], UV resistance [67,221] and so on, with the experimental results of these applications shown sequentially in Figure 14.

In the field of microfluidics, micro-droplets have important applications in areas such as drug release, virus detection and catalysts due to their small volume, large surface area, fast speed, high throughput, and uniform size [222,223]. Superhydrophobic surfaces, with their extremely small roll-off angles, can achieve efficient manipulation of droplets by designing a directional superhydrophobic surface so that the droplet can have a small roll-off angle in a specific direction [92,224], or by adjusting the contact angles at specific locations on the surface to make the droplet come into contact with the surface at selected positions [225].

In the field of food packaging, such as milk containers, residue inside the container during pouring will lead to food waste. Edible wax materials are treated and sprayed on the surface of elastic films, presenting a multiscale structure with wrinkles, resulting in extremely high contact angles for common beverages such as cola, milk and juice [226]. Beyond that, using magnetic particles to form a multiscale nano/micro-structured conical array on the surface of PDMS, with edible wax vapor sprayed on it, can achieve complete pouring of yogurt inside the container [86].

## 4. Conclusions

This paper offers a comprehensive summary of recent research on superhydrophobic modification of non-metallic material surfaces using multiscale nano/micro-structures. It mainly introduces various preparation methods, including the sol–gel method, molding method, etching method, deposition method, etc. Moreover, a summary is provided for the basic principles, preparation processes, research status, and advantages and disadvantages of these methods. It also shows the practical applications of superhydrophobic modification of non-metallic surfaces, such as anti-icing, oil–water separation, self-cleaning, etc. All of these have excellent application prospects by utilizing the performance advantages of superhydrophobicity according to specific needs.

Although superhydrophobic surfaces with multiscale nano/micro-structures have better durability than those with single-level nano-structures, the performance may decline over time, especially when encountering harsh environments such as dusty weather, extremely low temperatures and high-humidity conditions. Moreover, the preparation of multiscale nano/micro-structured surfaces generally requires multiple steps, posing challenges for the reproducibility of the process, and consequently hindering future large-scale applications in the engineering field. Hence, future key research priorities of non-metallic multiscale superhydrophobic surfaces for practical applications should focus on material designs with high durability and preparation techniques that are easy, easily scalable, effective and cost-efficient. The prospect of the preparation of multiscale nano/micro-structures also involves the integration of multiple disciplines, including materials science, nano-technology, surface science and other fields, which will provide us with a deeper understanding and means to control the surface properties of materials. This will allow non-metallic materials to play a more important role in scientific research and industrial applications, laying a solid foundation for future technology and innovation.
